# At home and away: A mobile transcription factor regulates meristem development in discrete spatial domains

**DOI:** 10.1093/plphys/kiac453

**Published:** 2022-09-26

**Authors:** Amy Lanctot

**Affiliations:** Cold Spring Harbor Laboratory, Howard Hughes Medical Institute, Cold Spring Harbor, New York 11724, USA

The shoot apical meristem (SAM) organizes all above-ground plant growth, regulating the formation of leaves and shoots during vegetative growth and then transitioning to flower formation during reproduction. For plants to continue to form new organs, the SAM must maintain a pool of stem cells in the central zone (CZ) while allowing for organogenesis to occur from cells derived from this undifferentiated pool in the peripheral zone (PZ; Ha et al., 2010). Consequently, SAM development requires complex feedback between cell division within the stem cell niche and differentiation in developing primordia.

Interplay between the hormones auxin and cytokinin establishes this feedback as each hormone promotes opposing fate states, with cytokinin promoting cell division and auxin promoting differentiation (Cheng et al., 2013). AUXIN RESPONSE FACTOR 3 (ARF3), a transcription factor that mediates auxin response, is a key regulator of organogenesis in the PZ. As *ARF3*’s own expression is promoted by auxin signaling, its expression pattern in the SAM closely mimics the distribution of auxin maxima in the developing primordia of the PZ (Sessions et al., 1997). ARF3 promotes primordia development by directly binding to and repressing the expression of cytokinin biosynthesis and response genes as well as key differentiation-repressing genes, such as *SHOOT MERISTEMLESS* (*STM*; Chung et al., 2019).

In this issue of *Plant Physiology*, Zhang et al. (2022) determine that ARF3 not only acts in the PZ, where the protein is synthesized, to promote organogenesis but also translocates to the CZ where it plays a role in SAM stem cell maintenance. The authors had previously shown that ARF3 protein localization does not match *ARF3* expression patterns, as the protein is present throughout the SAM, while the mRNA is restricted to the PZ (Liu et al., 2014). The authors hypothesized that ARF3 represses cell division to maintain stem homeostasis in the CZ. To test this hypothesis, they generated two ARF3 translational reporters, one with a nuclear localization sequence (NLS) that prevented ARF3 from moving outside the nucleus and consequently between cells. Localization of the ARF3-NLS reporter mimicked that of *ARF3* mRNA, suggesting that the NLS tag was disrupting ARF3 cell–cell movement.

**Figure 1:**
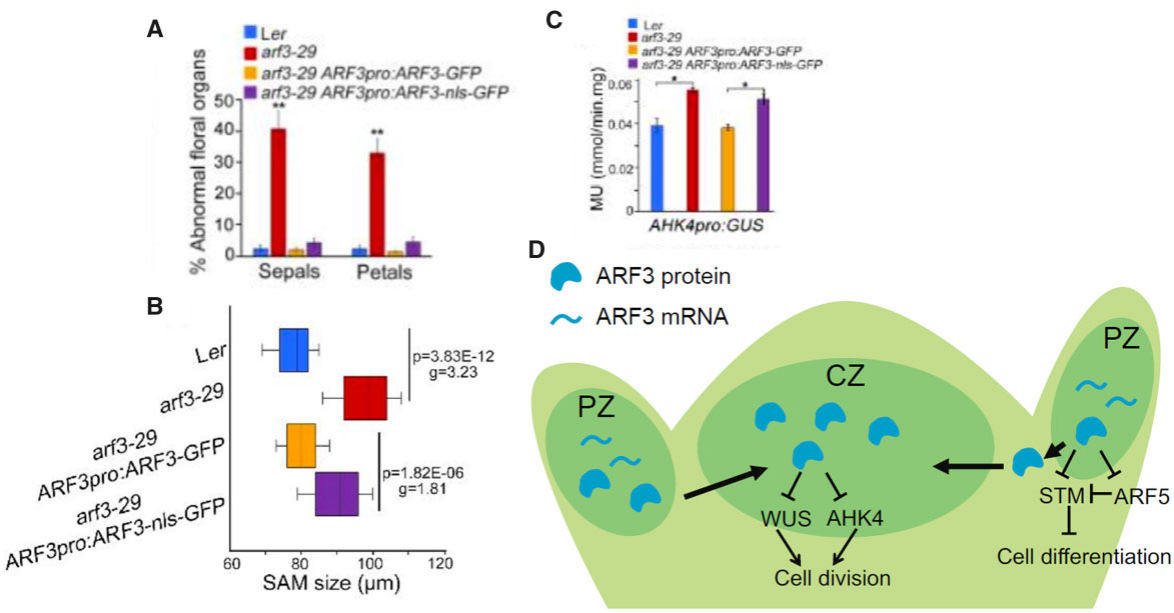
ARF3 mediates organogenesis and stem cell homeostasis in spatially discrete regions of the SAM. A, Quantification of flowers with abnormal numbers of sepals and petals in wild-type Arabidopsis (accession *Ler*), *arf3-29* mutant plants, and *arf3-29* mutants rescued with a mobile (ARF3pro:ARF3-GFP) and immobile (ARF3pro:ARF3-nls-GFP) translational reporter. B, Quantification of SAM size in wild-type Arabidopsis (accession *Ler*), *arf3-29* mutant plants, and *arf3-29* mutants rescued with a mobile (ARF3pro:ARF3-GFP) and immobile (ARF3pro:ARF3-nls-GFP) translational reporter. C, Quantification of WUS (WUSpro:DsRED) and AHK4 (AHK4pro:GUS) transcriptional reporter levels in wild-type Arabidopsis (accession *Ler*), *arf3-29* mutant plants, and *arf3-29* mutants rescued with a mobile (ARF3pro:ARF3-GFP) and immobile (ARF3pro:ARF3-nls-GFP) translational reporter. MU:mmol/min.mg. D, Schematic of ARF3 distribution, mobility, and signaling in the SAM. ARF3 is transcribed and translated in the PZ, where it downregulates expression of ARF5 and STM, the latter of which represses cell differentiation. The ARF3 protein moves to the CZ, where it downregulates expression of WUS and AHK4 to repress cell division. Figures adapted from Zhang et al. (2022), details of methods available in original figure legends.

The authors then tested whether the cell–cell mobile and immobile ARF3 translational reporters could rescue different *arf3* mutant phenotypes. Both reporters rescued *arf3* defects in organ initiation and floral organ number (Figure 1A), suggesting that migration of ARF3 from where it is transcribed in the PZ to the CZ is not required for organogenesis. Supporting this data, expression levels of an ARF3 target gene that represses *STM*, *AUXIN RESPONSE FACTOR 5* (*ARF5*; Chung et al., 2019), was fully rescued by both reporters. Furthermore, both reporters were shown through ChIP to bind to direct ARF3 target genes in the PZ. Phyllotactic patterning of flowers along the stem was also rescued by both reporters, suggesting that timing of organogenesis does not depend on ARF3 protein movement. In summary, ARF3-dependent developmental pathways that promote organ formation in the PZ do not rely on ARF3 protein mobility.

Conversely, ARF3-dependent developmental pathways that promote stem cell maintenance *do* rely on protein mobility. Arabidopsis (*Arabidopsis thaliana*) *arf3* mutants show enlarged SAMs, and these meristem size defects were fully rescued by the mobile reporter, with meristem size of the immobile NLS-tagged reporter being intermediate between the mutant and wild type (Figure 1B). Additionally, double mutants of *arf3* and the meristem-expressed gene *agamous* have strong defects in floral meristem development—these defects were fully rescued by the mobile reporter but only partially rescued by the immobile reporter. ARF3 downregulates expression of the cytokinin receptor *ARABIDOPSIS HISTIDINE KINASE 4* (*AHK4*) and the transcription factor *WUSCHEL* (*WUS*), both of which promote meristem proliferation in the CZ. Overexpression of *AHK4* and *WUS* in *arf3* mutants was rescued by the mobile reporter but not by the immobile one (Figure 1C), suggesting a possible mechanism by which ARF3 regulates SAM size.

This compelling work shows that ARF3 plays a role in both differentiation and division in the SAM, and the coordination of these two functions depends on the translocation of ARF3 protein from its site of transcription and translation in the PZ to the stem cell niche in the CZ (Figure 1D). In the PZ ARF3 represses expression of differentiation-repressing genes, consequently promoting differentiation and floral primordia development, and this function is not dependent on protein mobility. In the CZ, ARF3 represses expression of division-promoting genes to act as a check on cell proliferation and maintain meristem size and stem cell homeostasis. Maintaining the balance between cell division and differentiation is a key aspect of SAM development, and having a single molecular player act on both sides of this balance may simplify the transcriptional regulation of this homeostasis. The movement of ARF3 protein from the PZ to the CZ remains an intriguing area of research—what signals ARF3 to move from the PZ to the stem cell niche? Interestingly, WUS protein movement is a key component of WUS regulation in the SAM, as it travels to neighboring cells to repress expression of its own repressor *CLAVATA3* (Yadav et al., 2011). It may be that protein mobility is a common strategy to regulate complex spatial feedback in meristems.

It would also be interesting to further establish the role of feedback within auxin signaling on ARF3 mobility and function. ARF3 promotes auxin response, and its own expression is auxin induced. The authors show that ARF3 downregulates *ARF5* expression, another transcription factor that controls auxin response. ARF5 and ARF3 both repress expression of *STM* to promote differentiation, but ARF3 represses both *ARF5* and *STM* expression, generating a canonical incoherent feedforward loop (Alon, 2007) that allows for complex regulation of *STM* expression and consequently differentiation in the PZ. The interplay between different ARFs is an active area of research (Roosjen et al., 2018), but has been done mostly at the level of transcriptional regulation. Exploring how protein levels impinge upon the regulation of these complex circuits may further our understanding of homeostasis within the SAM, as this article shows that protein translocation is essential to consider when exploring these circuits.

*Conflict* *of interest statement.* None declared.
